# 14-3-3ζ suppresses RANKL signaling by destabilizing TRAF6

**DOI:** 10.1016/j.jbc.2024.107487

**Published:** 2024-06-21

**Authors:** R. Ayyasamy, S. Fan, P. Czernik, B. Lecka-Czernik, S. Chattopadhyay, R. Chakravarti

**Affiliations:** 1 Department of Physiology & Pharmacology, College of Medicine & Life Sciences, University of Toledo, Toledo, Ohio, USA; 2 Department of Medical Microbiology & Immunology, College of Medicine & Life Sciences, University of Toledo, Toledo, Ohio, USA; 3 Department of Orthopedics, College of Medicine & Life Sciences, University of Toledo, Toledo, Ohio, USA; 4 Microbiology, Immunology & Molecular Genetics, University of Kentucky College of Medicine, Lexington, Kentucky, USA

**Keywords:** 14-3-3ζ, YWHAZ, RANKL, TRAF6, osteoclast, bone homeostasis, macrophages, protein degradation, ubiquitin

## Abstract

Macrophages are essential regulators of inflammation and bone loss. Receptor activator of nuclear factor-κβ ligand (RANKL), a pro-inflammatory cytokine, is responsible for macrophage differentiation to osteoclasts and bone loss. We recently showed that 14-3-3ζ-knockout (*Ywhaz*^KO^) rats exhibit increased bone loss in the inflammatory arthritis model. 14-3-3ζ is a cytosolic adaptor protein that actively participates in many signaling transductions. However, the role of 14-3-3ζ in RANKL signaling or bone remodeling is unknown. We investigated how 14-3-3ζ affects osteoclast activity by evaluating its role in RANKL signaling. We utilized 14-3-3ζ-deficient primary bone marrow–derived macrophages obtained from wildtype and *Ywhaz*^KO^ animals and RAW264.7 cells generated using CRISPR-Cas9. Our results showed that 14-3-3ζ-deficient macrophages, upon RANKL stimulation, have bigger and stronger tartrate-resistant acid phosphatase–positive multinucleated cells and increased bone resorption activity. The presence of 14-3-3ζ suppressed RANKL-induced MAPK and AKT phosphorylation, transcription factors (NFATC1 and p65) nuclear translocation, and subsequently, gene induction (*Rank*, *Acp5*, and *Ctsk*). Mechanistically, 14-3-3ζ interacts with TRAF6, an essential component of the RANKL receptor complex. Upon RANKL stimulation, 14-3-3ζ–TRAF6 interaction was increased, while RANK–TRAF6 interaction was decreased. Importantly, 14-3-3ζ supported TRAF6 ubiquitination and degradation by the proteasomal pathway, thus dampening the downstream RANKL signaling. Together, we show that 14-3-3ζ regulates TRAF6 levels to suppress inflammatory RANKL signaling and osteoclast activity. To the best of our knowledge, this is the first report on 14-3-3ζ regulation of RANKL signaling and osteoclast activation.

The 14-3-3ζ is an adaptor protein known to interact with several cytokine receptors and MAPK (1, 2, 3). We recently showed that 14-3-3ζ suppresses inflammatory arthritis (IA) in animals (4). In the IA models, 14-3-3ζ-deficient (*Ywhaz*^KO^) rats show severe bone loss across several joints, affecting both cortical and trabecular compartments. These animals show decreased bone mass, cortical thickness, and trabeculae thickness with increased spacing between them. This suggested that 14-3-3ζ plays a role in maintaining bone homeostasis under inflammatory conditions. Bone remodeling is a coupled process involving bone formation by osteoblasts and bone resorption by osteoclasts (5). Uncoupling between anabolic and catabolic mechanisms can lead to a decreased bone mass, which is often seen in inflammatory diseases (6). IA is associated with an increase in osteoclasts' number and activity. Osteoclasts precursors of monocytic lineage fuse to make multinucleated and TRAP (tartrate-resistant acid phosphatase)-positive, mature osteoclasts in the presence of receptor activator of nuclear factor-κβ ligand (RANKL) (7, 8). RANKL levels are increased in IA-affected patients (9, 10, 11).

RANKL is an inflammatory cytokine secreted from osteocytes, osteoblasts, and T-cells (8). RANKL signals *via* its receptor RANK, which is a member of the TNF receptor family. It is a transmembrane protein present in the macrophages or pre-osteoclasts. RANK forms complexes with several adaptors (TRAF6, Grb2, and RACK1) and kinases (TAK, aPKC, and TAB1) in the cytosol for successful signal transduction (12). Upon binding RANKL, the cytoplasmic domain of RANK interacts with TRAF adaptor proteins, primarily TRAF2, TRAF5, and TRAF6, the E3 ubiquitin ligases (8). Among these, TRAF6 is essential for RANKL signaling and osteoclast activity. Traf6^KO^ mice, similar to RANK^KO^ or RANKL^KO^, show osteopetrosis, indicating its role in bone resorption and osteoclast function (13). Relatively less is known about other TRAFs (TRAF2 and TRAF5) (14). Activated RANK–TRAF complex induces a series of phosphorylation events involving MAPK, PI3K/AKT, and IκB. Activation of ERK and JNK leads to increased expression of c-Fos and c-Jun. At the same time, p38 phosphorylation promotes the expression of microphthalmia-associated transcription factor and TRAP. The activation of the PI3K-AKT pathway also supports osteoclast survival and function (15). RANKL induces NF-κB signaling by both classical and alternative pathways (16). Activation of the abovementioned transcription factors leads to the induction of several genes, such as *ACP5* (TRAP), *RANK*, *IL1b* (IL-1β), *MMP9*, *CTSK* (Cathepsin K), *etc.*, that are markers of osteoclast differentiation and activity (12). Several regulatory mechanisms of RANKL signaling help maintain balance between bone formation and bone resorption during remodeling. Like RANKL, osteoblasts also secrete osteoprotegerin, which acts as a soluble decoy receptor for RANKL, thus resulting in decreased RANKL-RANK signaling and osteoclast formation (8, 12). Signaling modulators (*e.g.* TAK1) or other factors (melatonin and bone morphogenetic proteins influence RANKL signaling and osteoclast differentiation (17, 18, 19). Several protein degrading pathways, such as autophagy and proteasomal degradation, play important role in RANKL signal transduction and osteoclastogenesis (20, 21).

We previously showed that 14-3-3ζ regulates IL-17A signaling (22). Like RANK, the cytoplasmic domain of IL-17A receptors (RA and RC) form complexes with TRAF (2, 5, and 6) proteins (23). In IL-17A signaling, 14-3-3ζ interacts and regulates TRAF-dependent outcomes (22). These results made us question if 14-3-3ζ′s role in bone remodeling is mediated by regulating RANKL signaling outcomes *via* TRAF proteins. We performed a detailed investigation to examine the biochemical mechanism of 14-3-3ζ-mediated regulation of RANKL signaling using genetically modified murine macrophage cell line [RAW264.7(RAW, henceforth] and rat bone marrow–derived primary macrophages (BMDMs). Our results, as shown below, indicate a novel role of 14-3-3ζ in suppressing RANKL signaling by promoting TRAF6 degradation. To the best of our knowledge, this is the first report of 14-3-3ζ regulation of RANKL signaling by affecting TRAF6 ubiquitination and stability.

## Results

### 14-3-3ζ suppresses RANKL-induced osteoclast differentiation

**Figure 1:**
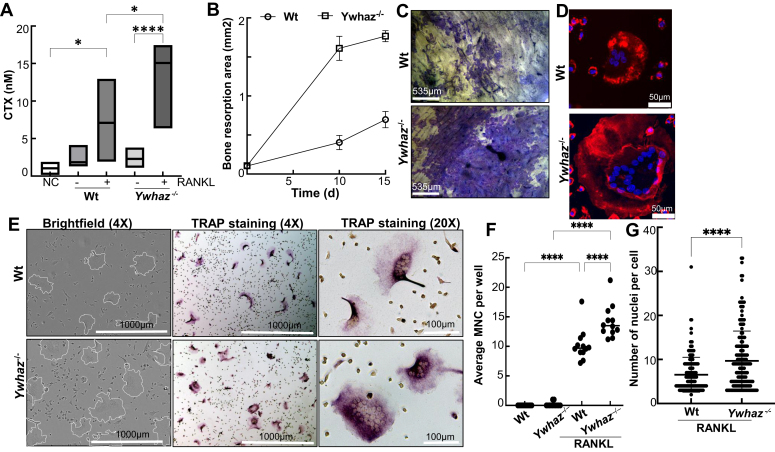
**14-3-3ζ suppresses RANKL-induced osteoclast formation and activity**. *A*, CTX levels in the conditioned media from 10 days post-RANKL-treated Wt and *Ywhaz*^KO^ BMDM plated on dentine discs were measured using ELISA (n = 2). *B* and *C*, primary BMDMs from Wt and *Ywhaz*^KO^ rats were plated on bone discs for 0 to 15 days with RANKL (100 ng/ml) followed by Toluidine blue staining. The pit area was quantified using ImageJ. Representative images from 10 days posttreatment are shown (n = 2). *D*, primary BMDMs from Wt and *Ywhaz*^KO^ animals at 4 days post RANKL (100 ng/ ml) treatment were stained with Texas red phalloidin to stain F-actin. The scale bar shows 50 μm (n = 2). *E*, primary BMDMs from Wt and *Ywhaz*^KO^ animals were treated with RANKL (100 ng/ml) and examined for multinucleated cells and TRAP staining at 4days. *White outlines* of multinucleated cells are shown on the brightfield image. A scale bar of 1000 μm or 100 μm is indicated on the images captured at 4× or 20×, respectively*. F* and *G*, over 150 cells from 25 microscopic fields of TRAP-positive multinucleated (3 nuclei or more) cells (captured at 4× magnification) were manually counted for an average number of MNC per well and number of nuclei per MNC. ∗∗∗∗ indicate *p* < 0.001. The experiment was performed thrice. BMDM, bone marrow–derived primary macrophage; MNC, multinucleated cell; RANKL, receptor activator of nuclear factor-κβ ligand; TRAP, tartrate-resistant acid phosphatase.

To understand why *Ywhaz*^KO^ animals show increased bone loss, we examined RANKL-induced osteoclastogenesis in wildtype (Wt) and *Ywhaz*^KO^ BMDMs. First, we measured the effect of 14-3-3ζ on osteoclast activity by plating Wt and *Ywhaz*^KO^ BMDMs on the dentine discs in the presence of RANKL and measuring collagen-type 1 fragments released in conditioned media using Crosslaps for Culture (CTX-I) assay. Incubating bone slices in media, without (NC) or with cells but no RANKL, showed minimum to little release of collagen products. RANKL treatment significantly increased collagen fragment (CTX) levels for the Wt cells. Compared to Wt, conditioned media from the 10 days post-RANKL–treated *Ywhaz*^KO^ BMDM showed higher CTX levels, indicating increased bone resorption in the absence of 14-3-3ζ (Fig. 1*A*). The dentine discs were stained with toluidine blue at 10 days and 15 days post-RANKL treatment, and the bone resorption area was measured. The discs plated with *Ywhaz*^KO^ BMDMs showed increased pit formation and corresponding bone resorption area over the tested period (Figs. 1, *B* and *C* and S1*A*). In contrast, the absence of RANKL did not cause any increase in the pit area for Wt or *Ywhaz*^KO^ BMDMs (Fig. S1*A*).

We next studied 14-3-3ζ′s role in the actin ring formation upon RANKL-treatment. Compared to Wt, RANKL treatment of *Ywhaz*^KO^ BMDMs resulted in much bigger actin rings visible as early as 4 days posttreatment (Figs. 1*D* and S1*B*). It was noted that the *Ywhaz*^KO^ BMDMs without RANKL treatment also showed the presence of an actin ring but not multinucleated cells (MNCs) (Fig. S1*B*). Next, we examined how 14-3-3ζ affects MNCs and TRAP staining, a marker of mature osteoclasts. Compared to Wt, RANKL-treated *Ywhaz*^KO^ BMDMs showed giant multinucleated and strongly TRAP-positive cells (Fig. 1*E*). The number of giant MNCs (MNC > 3 nuclei) over five fields (at 4× magnification) per well was counted and averaged for each treatment performed in quadruplicate. The average number of TRAP-positive MNC per well and the number of nuclei per MNC for *Ywhaz*^KO^ BMDMs was significantly higher than the Wt (Fig. 1, *F* and *G*). These results suggest that 14-3-3ζ suppresses RANKL-induced osteoclast differentiation and activity.

### 14-3-3ζ suppresses RANKL-mediated gene induction

**Figure 2:**
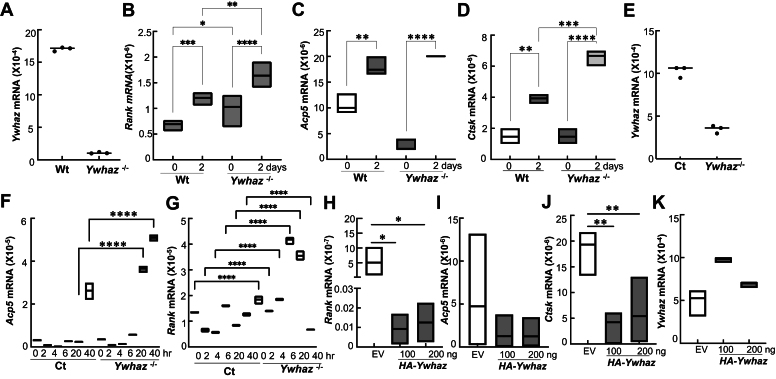
**14-3-3ζ suppresses RANKL-induced genes in macrophages**. *A*, *Ywhaz* mRNA levels in primary BMDMs obtained from Wt and *Ywhaz*^KO^ rats were analyzed by qRT-PCR (n = 2). *B–D*, mRNA levels of *Rank*, *Acp5*, and *Ctsk* in the M-CSF/RANKL-treated rat BMDMs were quantified by qRT-PCR (n = 2). *E*, *Ywhaz* mRNA levels in CRISPR-Cas9 control (Ct) and *Ywhaz*^KO^ RAW cells were analyzed by qRT-PCR. *F* and *G*, mRNA level of *Rank* and *Acp5* from the M-CSF/RANKL-treated RAW cells was measured by qRT-PCR (n = 3). *H–J*, effect of rescuing *Ywhaz*^KO^ BMDMs with different amounts of HA-14-3-3ζ with empty vector (EV) as control, was examined on 2 days post RANKL treatment on gene induction by qRT-PCR (n = 2). *K*, *Ywhaz* mRNA levels in rescued BMDM were analyzed by qRT-PCR. The expression levels of the mRNAs were normalized to 18S rRNA. BMDM, bone marrow–derived primary macrophage; RANKL, receptor activator of nuclear factor-κβ ligand; RAW, RAW264.7. The asterisks ∗, ∗∗, ∗∗∗, and ∗∗∗∗ show *p*-value of <0.05, 0.01, 0.005, and 0.001, respectively.

To understand how 14-3-3ζ regulates osteoclast differentiation, we compared RANKL-induced gene induction in the Wt and *Ywhaz*^KO^ cells. As expected for Wt BMDMs, RANKL stimulation caused an increase in *Rank*, *Acp5* (TRAP), and *Ctsk* mRNA levels. Compared to Wt, *Rank*, *Acp5*, and *Ctsk* mRNA levels were significantly increased in the *Ywhaz*^KO^ BMDMs (Fig. 2, *A*–*D*). Since RAW is a well-established model to investigate RANKL signaling, we generated 14-3-3ζ-deficient (*Ywhaz*^KO^) RAW cells using CRISPR/Cas9 (Fig. 2*E*) and examined its effect on RANKL-induced genes. Control CRISPR-Cas9 plasmid-transfected cells (labeled as Ct) were used as a control. A time course of gene induction upon RANKL stimulation was performed. Compared to Ct, *Ywhaz*^KO^ cells induced higher *Acp*5 and *Rank* mRNA levels (Fig. 2, *F* and *G*). To confirm the 14-3-3ζ role, we ectopically expressed epitope (HA)-tagged 14-3-3ζ (*HA-Ywhaz*) in *Ywhaz*^KO^ BMDMs and repeated gene induction study upon RANKL treatment. Compared to empty vector (EV), ectopic *HA-Ywhaz* expression suppressed RANKL-induced expression of *Rank*, *Acp5*, and *Ctsk* in *Ywhaz*^KO^ BMDMs (Fig. 2, *H*–*K*). These results indicated that 14-3-3ζ suppressed RANKL-induced gene expression.

### 14-3-3ζ suppresses RANKL-activated NF-κB and NFATC1

**Figure 3:**
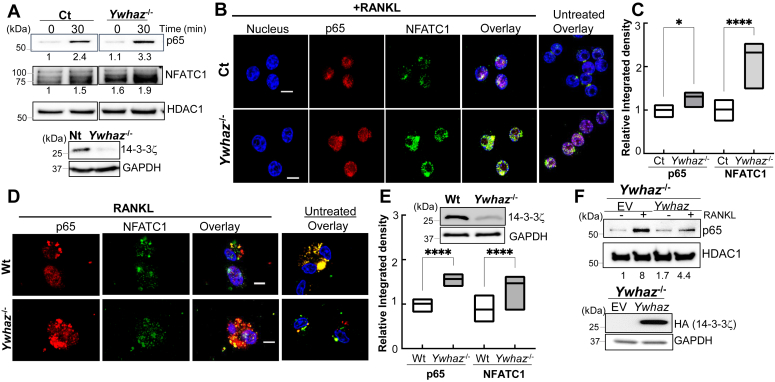
**14-3-3ζ suppresses RANKL-induced NF-κB and NFATC1 activation in macrophages**. *A*, Ct (CRISPR-Cas9 control) and *Ywhaz*^KO^ RAW cells were treated with M-CSF for 30 min, followed by RANKL for the indicated time. Nuclear fractions from the cells were analyzed for p65 and NFATC1 levels by immunoblot (n = 3). HDAC1 was used as a nuclear marker. Band intensities were quantified by ImageJ and are listed below each lane. 14-3-3ζ protein expression in Ct *versus Ywhaz*^KO^ RAW cells is shown in the lower panel. *B*, Ct and *Ywhaz*^KO^ RAW cells were treated with RANKL for 30 min when NFATC1 (*green*) and p65 (*red*) nuclear translocation (DAPI, *blue*) were analyzed by confocal microscopy. Overlays from untreated cells are shown (n = 2). *C*, ImageJ quantification of average nuclear fluorescence from 3 to 5 fields was calculated. *D*, primary BMDMs from Wt and *Ywhaz*^KO^ animals were treated with RANKL for 30 min, when NFATC1 (*green*) and p65 (*red*) nuclear translocation were analyzed by confocal microscopy. DAPI-stained nuclei are also shown in overlay images (n = 2). *E*, ImageJ quantification of average nuclear fluorescence from 3 to 5 fields was calculated. Loss of 14-3-3ζ at the protein level is shown. *F*, the *Ywhaz*^KO^ RAW cells were transiently transfected with either an empty vector (EV) or HA-*Ywhaz* and stimulated with RANKL for 30 min. Nuclear fractions were examined for p65 nuclear translocation by immunoblot (n = 3). Expression of HA-14-3-3ζ is shown by immunoblot using anti-HA antibody. Molecular weights are shown on the *left* of the immunoblot panels. Original blots are shown in the supplementary data. BMDM, bone marrow–derived primary macrophage; RANKL, receptor activator of nuclear factor-κβ ligand; RAW, RAW264.7. The asterisks ∗ and ∗∗∗∗ show *p*-value of <0.05 and 0.001, respectively.

RANKL-stimulated nuclear translocation of several transcription factors, including p65, NFATC1, and c-Jun, was evaluated in the Ct and *Ywhaz*^KO^ cells using subcellular fractionation. Nuclear translocation of NF-κB subunit p65 and NFATC1 was increased in RANKL-stimulated *Ywhaz*^KO^ compared to the Ct RAW cells. (Figs. 3*A* and S2*A*). No significant changes in the c-Jun levels were observed between the Ct and *Ywhaz*^KO^ cells (not shown). We further confirmed these results by confocal microscopy, which indicated increased signals for nuclear p65 and NFATC1 in the RANKL-treated *Ywhaz*^KO^ cells compared to control cells (Fig. 3, *B* and *C*). It was noted that *Ywhaz*^KO^ cells showed increased nuclear levels of p65 and NFATC1 at the basal level, which was further increased upon RANKL treatment. Similar to RAW cells, increased nuclear levels of p65 and NFATC1 were also observed in the RANKL-treated primary *Ywhaz*^KO^ BMDMs (Fig. 3*D*). Similar to RAW cells, a statistically significant increase in the nuclear staining of p65 and NFATC1 was observed in the *Ywhaz*^KO^ BMDM (Fig. 3*E*). To confirm its role in regulating RANKL-induced nuclear localization of transcription factors, we rescued 14-3-3ζ in the *Ywhaz*^KO^ cells using transient transfections with an HA-tagged 14-3-3ζ expression vector. Compared to EV, rescue with *HA-Ywhaz* increased 14-3-3ζ and suppressed nuclear p65 levels upon RANKL stimulation (Figs. 3*F* and S2*B*). These results indicate that 14-3-3ζ suppressed RANKL-mediated NF-κB and NFATC1 activation.

### 14-3-3ζ suppresses RANKL-induced MAPK and AKT phosphorylation

**Figure 4:**
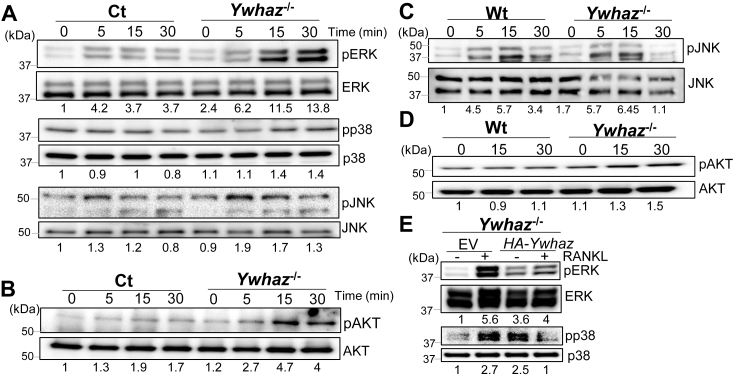
**14-3-3ζ suppresses RANKL-induced MAPK and AKT phosphorylation**. *A* and *B*, Ct and *Ywhaz*^KO^ RAW cells were pretreated with M-CSF for 30 min, followed by RANKL treatment for the indicated time, when phosphorylation of ERK, p38, JNK, and AKT were analyzed by immunoblot (n = 3). Band intensities of the phosphoproteins, normalized to their respective total protein levels, quantified by Image J, are shown under each lane*. C* and *D*, Wt and *Ywhaz*^KO^ primary BMDMs were pretreated with M-CSF for 30 min, followed by RANKL treatment for the indicated time, when phosphorylation of JNK and AKT were analyzed by immunoblot (n = 2). *E*, *Ywhaz*^KO^ knockout primary BMDMs, rescued with either empty vector (EV) or HA-*Ywhaz*, were examined for RANKL-induced phosphorylation of ERK and p38 by immunoblot (n = 2). Band intensities, quantified by Image J, are listed under each lane. 14-3-3ζ protein expression is shown in Fig. 2. Molecular weights are shown on the *left* of the representative Western blot panels. Original blots are shown in the supplementary data. BMDM, bone marrow–derived primary macrophage; RANKL, receptor activator of nuclear factor-κβ ligand; RAW, RAW264.7.

We further examined 14-3-3ζ′s role in RANKL-induced signaling in the Ct and *Ywhaz*^KO^ RAW cells. RANKL treatment of RAW (Ct) increased phosphorylation of several intermediate kinases, including MAPK (ERK, p38, and JNK) and AKT. In comparison, phosphorylation of all intermediate kinases (ERK, p38, JNK, and AKT) was more increased in the *Ywhaz*^KO^ RAW cells (Figs. 4, *A* and *B* and S3, *A* and *B*). Similarly, *Ywhaz*^KO^ primary BMDMs also showed increased phosphorylation of AKT and JNK when compared to Wt BMDMs (Figs. 4, *C* and *D* and S3, *C* and *D*). 14-3-3ζ rescued *Ywhaz*^KO^ BMDMs, compared to EV, show reduced levels of RANKL-induced ERK and p38 phosphorylation (Figs. 4*E* and S3*E*). Together, our results demonstrated that 14-3-3ζ suppressed RANKL signaling in macrophages.

### *14-3-3ζ–*TRAF6 interaction is increased by RANKL

**Figure 5:**
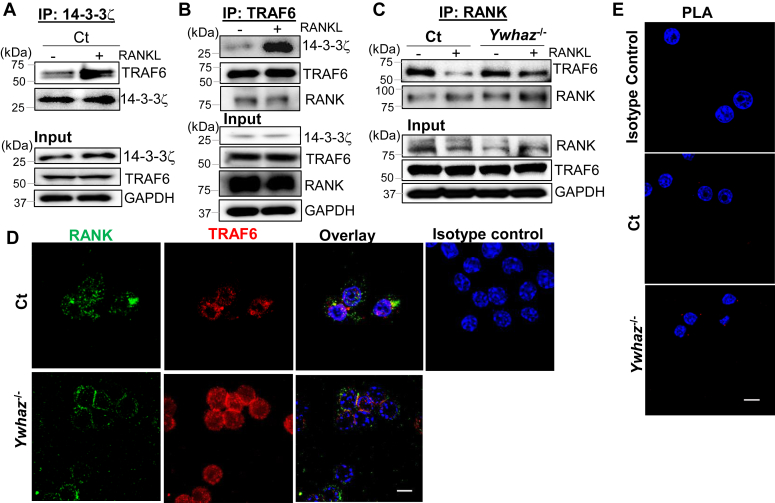
**14-3-3ζ targets TRAF6–RANK interaction to suppress RANKL signaling**. *A*, five minutes RANKL-treated Ct RAW cells were IP’ed with anti-14-3-3ζ IgG, and the elutes were immunoblotted for TRAF6. Individual protein expression in the input cell lysates is also shown (n = 3). *B*, co-IP of TRAF6 from RANKL-treated Ct cells were probed for 14-3-3ζ and RANK. Input cell lysates are also shown (n = 3). *C*, co-IP of RANK from RANKL-treated Ct and *Ywhaz*^KO^ RAW cells and analyzed for TRAF6 by immunoblot (n = 3). Original blots are shown in the supplementary data. *D*, immunostaining followed by confocal imaging (100×) shows RANK (*green*), TRAF6 (*red*), and DAPI (*blue*) in the Ct and *Ywhaz*^KO^ RAW cells. Isotype control in Ct cells is shown (n = 2). The scale bar shows 10 μm. *E*, confocal imaging of PLA shows TRAF6 and RANK interaction (*red dots*) in the RANKL-treated Ct and *Ywhaz*^KO^ cells (n = 2). The isotype control on Ct cells is shown. The scale bar shows 10 μm. co-IP, co-immunoprecipitation; PLA, proximal ligation assay; RANK, receptor activator of nuclear factor-κβ; RANKL, receptor activator of nuclear factor-κβ ligand; RAW, RAW264.7.

Results shown above suggest that 14-3-3ζ action lies upstream of signaling kinases (*e.g.*, MAPK). Therefore, we next examined 14-3-3ζ′s role at the receptor complex by performing protein–protein interaction studies immediately after RANKL stimulation. Co-immunoprecipitation (co-IP) studies in Ct RAW cells showed that 14-3-3ζ interacts with TRAF6, which further increased upon 5 min of RANKL stimulation (Figs. 5*A* and S4*A*). The 14-3-3ζ knockout cells were used as control (Fig. S4*B*). This result was further confirmed by co-IP of TRAF6, which again showed increased TRAF6–14-3-3ζ interaction in RANKL-treated cells (Figs. 5*B* and S4*C*). TRAF6–RANK interaction, a key event for RANKL signal transduction, was evaluated by co-IP of RANK and TRAF6 in Ct and *Ywhaz*^KO^ cells. Importantly, RANK and TRAF6 interacted in the absence of RANKL stimulation. Upon RANKL stimulation, the TRAF6–RANK interaction decreased in both Wt and *Ywhaz*^KO^ cells. However, TRAF6–RANK interaction remained significantly higher in RANKL-treated *Ywhaz*^KO^ than in Ct cells (Figs. 5*C* and S4*D*). Next, we performed immunostaining to examine TRAF6–RANK interaction in RAW cells. Confocal microscopy revealed that surface localization and overall staining of TRAF6 were visibly increased in the RANKL-treated *Ywhaz*^KO^ cells. This correlated with increased colocalization of TRAF6 and RANK, as shown by yellow-colored overlays (Fig. 5*D*). To confirm that 14-3-3ζ regulates TRAF6–RANK interaction, we performed a proximal ligation assay. Results showed increased interaction (red dots) between TRAF6 and RANK in the RANKL-treated *Ywhaz*^KO^ cells (Figs. 5*E* and S4*E*). These results suggested that 14-3-3ζ interacts with TRAF6 and suppresses TRAF6–RANK interaction in RANKL-treated cells.

### 14-3-3ζ promotes TRAF6 degradation upon RANKL stimulation

**Figure 6:**
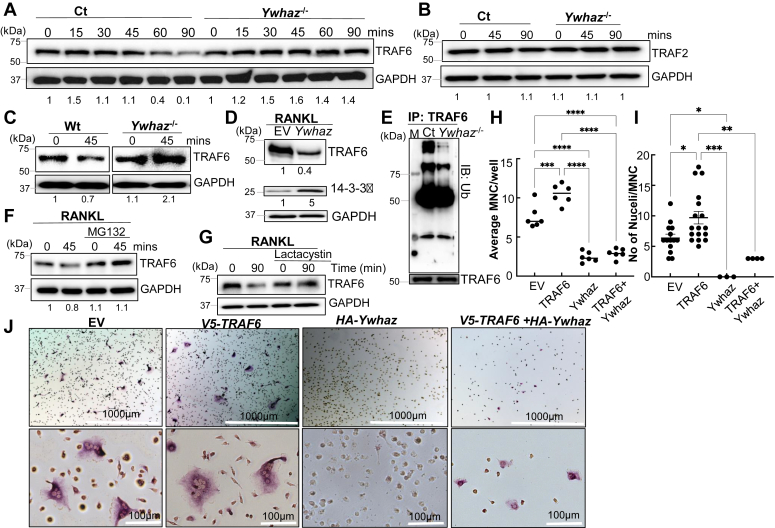
**14-3-3ζ promotes TRAF6 degradation upon RANKL stimulation**. *A*, TRAF6 levels were monitored over 90 min post-RANKL treatment in Ct and *Ywhaz*^KO^ RAW cells by immunoblot (n = 3). *B*, TRAF2 levels in RANKL-treated Ct and *Ywhaz*^KO^ RAW cells by immunoblot are shown (n = 3). *C*, primary BMDMs from Wt and *Ywhaz*^KO^ rats were cultured and treated with RANKL for 45 min to examine TRAF6 levels by immunoblot (n = 2). *D*, TRAF6 levels were compared in the 90-min post-RANKL treatment of *Ywhaz*^KO^ RAW cells rescued with *HA-Ywhaz* or empty vector (n = 3). *E*, TRAF6 was immunoprecipitated from RANKL-treated Ct and *Ywhaz*^KO^ RAW cells and analyzed for ubiquitination by immunoblot (n = 3). *F* and *G*, TRAF6 levels were monitored in RANKL-treated Ct RAW cells in the presence of MG132 and lactacystin by immunoblot (n = 3). *H–J*, the average number of MNC (>3 nuclei) per well and average number of nuclei per TRAP-positive multinucleated cell in the Wt BMDM transfected with EV, *V5*-*TRAF6*, and/or *HA-Ywhaz* for 48 h followed by 3 days RANKL treatment, was manually counted. An average of 10 fields were examined. The experiment was performed thrice. The representative TRAP-positive stained cells, imaged at 4× and 20× magnifications, are shown. BMDM, bone marrow–derived primary macrophage; MNC, multinucleated cell; RANKL, receptor activator of nuclear factor-κβ ligand; RAW, RAW264.7; TRAP, tartrate-resistant acid phosphatase. The asterisks ∗, ∗∗, ∗∗∗, and ∗∗∗∗ show *p*-value of <0.05, 0.01, 0.005, and 0.001, respectively.

We next questioned the consequence of 14-3-3ζ interference in RANK–TRAF6 interaction. Our imaging studies showed an increase in TRAF6 signal in *Ywhaz*^KO^ compared to Ct cells (Fig. 5*D*). RANKL-induced TRAF6 degradation has been shown to dampen downstream signaling (20). We performed a time course study to examine TRAF6 protein levels in the RANKL-treated Ct and *Ywhaz*^KO^ RAW cells. Over 90 min post-RANKL treatment, a significant reduction in TRAF6 protein levels was noted in the Ct cells but not in the *Ywhaz*^KO^ RAW cells (Figs. 6*A* and S5*A*). Reduction in TRAF6 levels in *Ywhaz*^KO^ RAW cells was RANKL dependent since no difference in TRAF6 levels was observed in unstimulated cells (Fig. S5*B*). Also, RANKL treatment in the presence of cycloheximide did not affect TRAF6 level, suggesting new protein synthesis did not play any role in this process (Fig. S5*C*). To determine the specificity of TRAF6 degradation, we examined the effect on TRAF2 levels, which did not change upon RANKL stimulation or by the presence of 14-3-3ζ (Figs. 6*B* and S5*D*). The 14-3-3ζ-dependent TRAF6 degradation was also observed in the RANKL-treated primary BMDMs from Wt and *Ywhaz*^KO^ animals (Fig. 6*C*). Importantly, the effect on TRAF6 stability was specific to RANKL, as M-CSF treatment did not affect TRAF6 levels (Fig. S5*E*). To confirm that 14-3-3ζ regulated TRAF6 levels, we restored 14-3-3ζ expression in the *Ywhaz*^KO^ RAW cells and examined RANKL-induced TRAF6 degradation. RANKL treatment of 14-3-3ζ-restored *Ywhaz*^KO^ RAW cells resulted in reduced TRAF6 levels (Figs. 6*D* and S5*F*).

Since proteasomal degradation has been shown to regulate TRAF6 (20), we examined if 14-3-3ζ has any role in regulating TRAF6 ubiquitination (Ub-TRAF6). The IP of TRAF6 followed by immunoblotting with anti-ubiquitin IgG showed that RANKL-induced Ub-TRAF6 level was decreased in the *Ywhaz*^KO^ cells (Figs. 6*E* and S5*G*). To confirm that proteasomal degradation is responsible for TRAF6 levels, we used MG132 and lactacystin, which suppressed RANKL-induced TRAF6 degradation in the Ct cells. These results indicated that 14-3-3ζ promoted RANKL-stimulated TRAF6 degradation *via* the proteasomal pathway, which can be prevented using MG132 (10 nM) and lactacystin (10 nM) (Figs. 6, *F* and *G* and S5, *H* and *I*). To examine the functional relevance of 14-3-3ζ and TRAF6 levels, we overexpressed TRAF6 or *HA-Ywhaz* in the Wt BMDMs and studied osteoclast differentiation using TRAP staining. Increased levels of TRAF6, compared to EV, resulted in more and bigger TRAP-positive MNCs upon RANKL stimulation. However, co-expression of 14-3-3ζ with TRAF6 in BMDM suppressed TRAF6’s promotional effect on RANKL-induced TRAP-positive MNC generation. Co-expression of 14-3-3ζ with an EV significantly suppresses TRAP-stained giant MNCs per well and the number of nuclei per cell upon RANKL treatment (Figs. 6, *H*–*J* and S5*J*).

**Figure 7:**
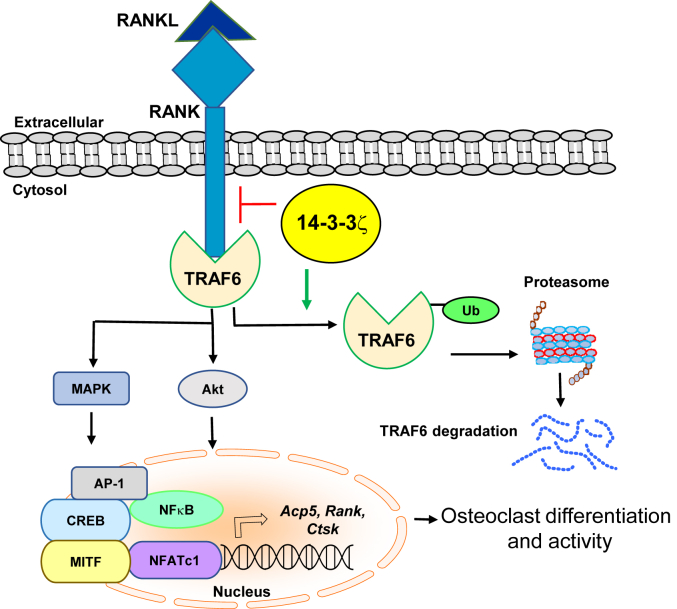
**14-3-3ζ promotes TRAF6 degradation to suppress RANKL signaling and osteoclastogenesis**. A schematic of 14-3-3ζ′s suppressive action on RANKL signaling and osteoclast activity interferes with TRA6–RANK interaction and promotes TRAF6 ubiquitination and degradation. This dampens the MAPK and AKT phosphorylation events and nuclear translocation of p65 and NFATC1, thus resulting in reduced induction of *Acp5*, *Rank*, and *Ctsk*, genes responsible for osteoclast differentiation and activity. RANKL, receptor activator of nuclear factor-κβ ligand; RANK, receptor activator of nuclear factor-κβ.

Together, our studies reveal that 14-3-3ζ suppresses RANKL-induced signal transduction, gene induction, and osteoclastogenesis by promoting TRAF6 degradation *via* the proteasomal pathway (Fig. 7).

## Discussion

RANKL signaling is primarily responsible for osteoclast activity and bone loss. Our results show that 14-3-3ζ is a suppressor of RANKL signaling in macrophages. By manipulating 14-3-3ζ levels, knockout in RAW cells and primary BMDM, as well as ectopically expression to rescue the levels, we demonstrated it suppresses RANKL-induced phosphorylation events of intermediate kinases, nuclear translocation of transcription factors, and target gene expression. Biochemical and imaging results showed that RANKL stimulation caused increased interaction for 14-3-3ζ-TRAF6 while decreased TRAF6–RANK interaction. The presence of 14-3-3ζ promoted TRAF6 ubiquitination and degradation during early time points post-RANKL treatment (Fig. 7). However, 14-3-3ζ did not affect TRAF6 levels in unstimulated cells or only M-CSF-treated cells, suggesting that the effect is RANKL dependent. Suppression of RANKL signaling by 14-3-3ζ was functionally visible by stronger and bigger TRAP-positive cells and actin rings in the *Ywhaz*^KO^ cells. This correlated with increased toluidine staining, pit formation, and release of collagen fragments in media, suggesting higher osteoclast activity of *Ywhaz*^KO^ cells. Though the absence of 14-3-3ζ at the basal level showed an actin ring, cells were neither multinucleated nor had any bone resorption activity. Overall, our results clearly indicate that 14-3-3ζ suppresses RANKL signaling and osteoclast activity.

14-3-3ζ is a member of the 14-3-3 protein family (24, 25). In addition to acting as adaptors, these are known to regulate the nuclear-cytoplasmic shuttling of several transcription factors. It has been shown that 14-3-3ζ participates in the nuclear translocation of NF-κB and microphthalmia-associated transcription factor (26, 27). Similarly, 14-3-3γ is reported to participate in the selenoprotein W–regulated shuttling of NFATC1 and NF-κB in RANKL signaling (28). Our results, on the contrary, indicate that 14-3-3ζ suppresses NF-κB and NFATC1 nuclear translocation in the RANKL signaling. We previously reported that 14-3-3ζ interacts with TRAF6 in fibroblasts and epithelial cells (22). Our current study extends this interaction to macrophages wherein TRAF6 interacts with the cytoplasmic domain of RANK (14, 23). TRAF6 has E3 ubiquitin ligase activity that is essential for RANKL signaling (29). The association and strength of TRAF6–RANK interaction play a key role in osteoclast differentiation (30, 31, 32). Our results showing that 14-3-3ζ reduces TRAF6 levels and weakens the RANK–TRAF6 interaction in RANKL-stimulated cells can explain its suppressive action on RANKL signaling. How 14-3-3ζ binds to TRAF6 has not been studied. The 14-3-3 proteins can bind both phosphorylated and nonphosphorylation proteins (33, 34). Phosphorylation of TRAF proteins, except for TRAF4, remains one of the least studied posttranslational modifications (35). In the LPS signaling, TRAF6 phosphorylation plays a regulatory role in ubiquitination and proteasomal degradation (36). Though TRAF6 interaction with atypical PKC and p62 in the RANKL signaling has been reported, how it affects TRAF6 phosphorylation remains unknown (37). Since 14-3-3ζ–TRAF6 interaction is increased upon RANKL stimulation; there is a possibility that a posttranslational modification or a third protein(s) may participate in this interaction. It is known that p62 promotes TRAF6 ubiquitination and NF-κB activity in RANKL signaling (37, 38, 39, 40). As an autophagy regulator, p62 connects 26S proteasomal degradation and autophagy pathways (39, 40, 41). It is known that 14-3-3ζ regulates autophagy in a context-dependent manner (42, 43). How 14-3-3ζ interacts with TRAF6 and regulates its degradation *via* proteasomal degradation or autophagy upon RANKL stimulation will be investigated in future studies.

TRAF proteins are essential in various pathways, including RANKL signaling (14, 44). In addition to TRAF6, TRAF2 and TRAF5 also interact with RANK; however, the site of TRAF6 interaction on RANK is different from TRAF2 and TRAF5 (14). TRAF6 is an E3 ubiquitin ligase, and its interaction with TAK1-TAB to promote RANKL signaling and osteoclastogenesis depends upon its ubiquitination status (29, 45). Removal of K63 ubiquitin chains on TRAF6 by CYLD, a de-ubiquitinase recruited by p62, suppresses RANKL signaling; however, CYLD involvement appears to play a significant role at the pre-osteoclast level but not during the early part of RANKL signaling (41, 45). In contrast to K63, K48-ubiquitination of TRAF6 promotes its degradation and suppresses RANKL signaling (46, 47). Apart from ubiquitination, additional mechanisms affect TRAF6 activity in RANKL signaling. Recently, Annexin 3 has been shown to promote TRAF6 stability and interaction with RANK to activate RANKL signaling (48). In contrast, WDFY3 regulates TRAF6 levels by autophagy to suppress RANKL signaling and osteoclastogenesis (21). Notably, several 14-3-3 isoforms, including the zeta, are known to control the cellular level of ubiquitinated proteins (49, 50, 51). Our results indicate that 14-3-3ζ promotes TRAF6 ubiquitination and degradation *via* the proteasomal pathway in RANKL-treated cells. Further investigations are needed to study if 14-3-3ζ-mediated TRAF6 ubiquitination is K48-linkage specific, which is known for protein degradation (52).

While several inflammatory cytokines (IL-17A and TNF-α) promote bone loss, RANKL is a primary cytokine responsible for osteoclastogenesis and bone loss (53, 54, 55). In addition to IA, RANKL levels are increased in several immune diseases, including primary biliary cholangitis (56), inflammatory bowel disease (57), and type 2 diabetes mellitus (58). Suppression of RANKL signaling and osteoclast activity by 14-3-3ζ supports *in vivo* results observed in the IA model (4). Based on our current and published studies demonstrating 14-3-3ζ′s role in regulating TRAF6-dependent IL-17A and RANKL signaling, we speculate that the 14-3-3ζ may have broader implications in other innate mechanisms requiring TRAF6. Overall, our results show that 14-3-3ζ is a novel suppressor of RANKL signaling and osteoclast activation.

## Experimental procedures

### Reagents

All chemicals were purchased from Fisher Scientific Inc. unless stated otherwise. Murine and rat M-CSF and RANKL were obtained from R&D Systems. CTX-I ELISA and dentine slices were purchased from immunodiagnostic systems, IDS. The antibodies against the specific proteins were purchased from CST unless indicated otherwise: phospho-ERK, ERK, phospho-AKT (Ser), AKT, phospho-p38, p38, phospho-JNK, anti-JNK, TRAF6, TRAF2, GAPDH, HDAC1, HA, p65, and 14-3-3ζ. The antibodies, including NFATc1, c-Jun, 14-3-3ζ, RANK, β-Tubulin, and CRISPR-Cas9 constructs of 14-3-3ζ (*Ywhaz*) and control (Ct) were purchased from Santa Cruz Biotechnology Inc.

### Cell culture

The RAW 264.7 cells were purchased from ATCC, and cells were maintained in Dulbecco's modified Eagle's medium containing 10% FBS, penicillin, and streptomycin at 37 ^◦^C and 5% CO_2_ incubators. The cells were pretreated with M-CSF (50 ng/ml) for 30 min, followed by mouse RANKL (50 ng/ml) treatment for the time as indicated later (59). The cleaned bones of Wt and *Ywhaz*^KO^ rats were used to collect primary BMDMs. The bone marrow was collected by spinning in a clean tube at 1000 rpm for 5 min. The red blood cells were lysed using ammonium-chloride-potassium lysis buffer, followed by plating in the minimum essential medium containing 20% LCM (L929 conditioned media), 15% fetal bovine serum, and 5 μM β-mercaptoethanol. After 1 day, nonadherent cells were collected and cultured on plastic dishes or glass coverslips with M-CSF (10 ng/ml) and RANKL (100 ng/ml) to induce osteoclast differentiation. The BMDMs were transfected with EV, V5-TRAF6, or HA-14-3-3ζ for 48h. Cells were treated with rat RANKL for the time period as indicated in figure legends.

The 14-3-3ζ knockout RAW cells were generated using the CRISPR-Cas9 method as described before (22). Briefly, RAW 264.7 cells were transfected with either control (sc-418922) or 14-3-3ζ–specific (sc-400490) CRISPR/Cas9 plasmids (Santa Cruz Biotechnology Inc) using Lipofectamine 2000 (Thermo Fisher Scientific). Transfected cells were sorted for high GFP-expressers using flow cytometry, and the GFP-expressing cells were expanded to isolate individual clones. These clones were screened for 14-3-3ζ protein levels by immunoblot, and the clones with no 14-3-3ζ protein expression were selected for further validation to ensure the gene knockout. Henceforth, the CRISPR-control plasmid-transfected cells are listed as Ct and 14-3-3ζ knockout as *Ywhaz*^KO^. For overexpression or rescue, cells were transfected with HA-14-3-3ζ or an EV for 48 h. The medium was then replaced with fresh complete Dulbecco's modified Eagle's medium/minimum essential medium containing M-CSF and RANKL for the time as needed for a specific experiment.

### TRAP staining

TRAP-positive cells were determined using a leukocyte acid phosphate assay kit (Sigma) as per the manufacturer’s instructions. The cells were fixed with 65% acetone, 25% citrate solution, and 8% formaldehyde. The fixed cells were then incubated with the TRAP staining solution in the dark for 1 h at 37 °C. After washing twice with water, the cells were counterstained with hematoxylin for 30 s and rinsed with water. TRAP-positive cells that contained three or more nuclei were considered mature osteoclasts when visualized under Cytation 5 (BioTek).

### Immunostaining

The RANKL-treated cells were fixed in 4% (v/v) paraformaldehyde and permeabilized with 0.1% Triton X-100 (v/v), as used before (22). For immunostaining, coverslips were blocked using 1% BSA following adding primary antibodies followed by Alexa Fluor-conjugated secondary antibody (Invitrogen Inc.). The previously optimized protocol for proximal-ligation assay was used (22). For actin staining, 100 μl of 2 μM Texas red phalloidin was added to the fixed and permeabilized cells for 30 min in the dark at room temperature (60). The coverslips were mounted onto microscopy slides using DAPI containing VectaShield (Vector Laboratories #H-1200) and analyzed using a Leica microscope and Las X software or SP5 Laser Scanning Confocal Microscope with MP (Leica Microsystems).

### C-terminal telopeptide fragments of type I collagen and resorption pits assay

BMDMs were plated on the washed bone slices and incubated with M-CSF and RANKL. At 10 days posttreatment, conditioned media were collected, and collagen degradation products using the CTX-I ELISA kit were quantified as per the manufacturer’s instructions (CrossLaps).

To identify the resorption pits formed on the dentine slices, attached cells were removed from the discs by sonication. The bone slices were washed and stained with toluidine blue (0.1%, w/v). Bone slices were imaged using a Leica microscope, and the total areas of resorption pits were determined using NIH Image-J software.

### Immunoblots and immunoprecipitation

Immunoblot analyses were performed using previously described procedures (22). Briefly, the cells were lysed in 50 mM Tris buffer, pH 7.4, containing 150 mM of NaCl, 0.1% Triton X-100, 1 mM sodium orthovanadate, 10 mM sodium fluoride, 10 mM β-glycerophosphate, 5 mM sodium pyrophosphate, and protease and phosphatase inhibitors (Roche). The 2 μg of antibody followed by Protein-G-Sepharose for co-IP or 10 μl of tagged beads for affinity pulldowns were added to the cell lysate and incubated overnight at 4 °C. Total protein extracts, or pull-down beads, were analyzed by SDS/PAGE, followed by immunoblot.

### RNA isolation and quantitative analysis

Total RNA was isolated using TRIzol (Invitrogen), cDNA was prepared using ImProm-II Reverse Transcription Kit (Promega), and the cDNA was analyzed using Radiant SYBR Green PCR mix (Alkali Scientific) in Roche LightCycler 96 instrument and analyzed with the LightCycler 480 Software, v1.5. The expression levels of the mRNAs were normalized to 18S rRNA. For the qRT-PCR analyses of the respective genes, the following primers were used:

rAcp5: CGCCAGAACCGTGCAGA/TCAGGCTGCTGGCTGAC

rRank: TTAAGCCAGTGCTTCACGGG/ACGTAGACCACGATGATGTCGC

rCtsk: CCCAGACTCCATCGACTATCG/CTGTACCCTCTGCACTTAGCTGCC

rYwhaz: TGAAGAGTCGTACAAAGACAGCA/GTTAATTTTCCCCTCCTTCTCC

mAcp5: GACGATGGGCGCTGACTTCA/GCGCTTGGAGATCTTAGAGT

mRank: TTTGTGGAATTGGGTCAATGAT/ACCTCGCTGACCAGTGTG AA

mCtsk: ACGGAGGCATCGACTCTGAA/GATGCC AAGCTTGCGTCGAT

mYwhaz: ACCGTTACTTGGCCGAGGTT/GCAGGCTTTCTCTGGGGAGT.

### Statistical analysis

All experiments with RAW cells and BMDM were performed at least thrice unless indicated otherwise. All imaging studies are performed thrice. GraphPad Prism was used to compare the number of sets by unpaired Student’s *t* test or two-way ANOVA. The asterisks ∗, ∗∗, ∗∗∗, and ∗∗∗∗ show *p*-value of <0.05, 0.01, 0.005, and 0.001, respectively.

## Data availability

All data presented in this paper are contained within the article.

## Supporting information

This article contains supporting information.

## Conflict of interest

The authors declare that they have no conflicts of interest with the contents of this article.
