# Supplementary material for: 14-3-3ζ suppresses RANKL signaling by destabilizing TRAF6
Source: J Biol Chem. 2024 Jun 21;300(7):107487. doi: 10.1016/j.jbc.2024.107487 (PMC11331427; doi:10.1016/j.jbc.2024.107487)
Supplement: Supporting Figures [file mmc1.pdf]

Supplementary Data

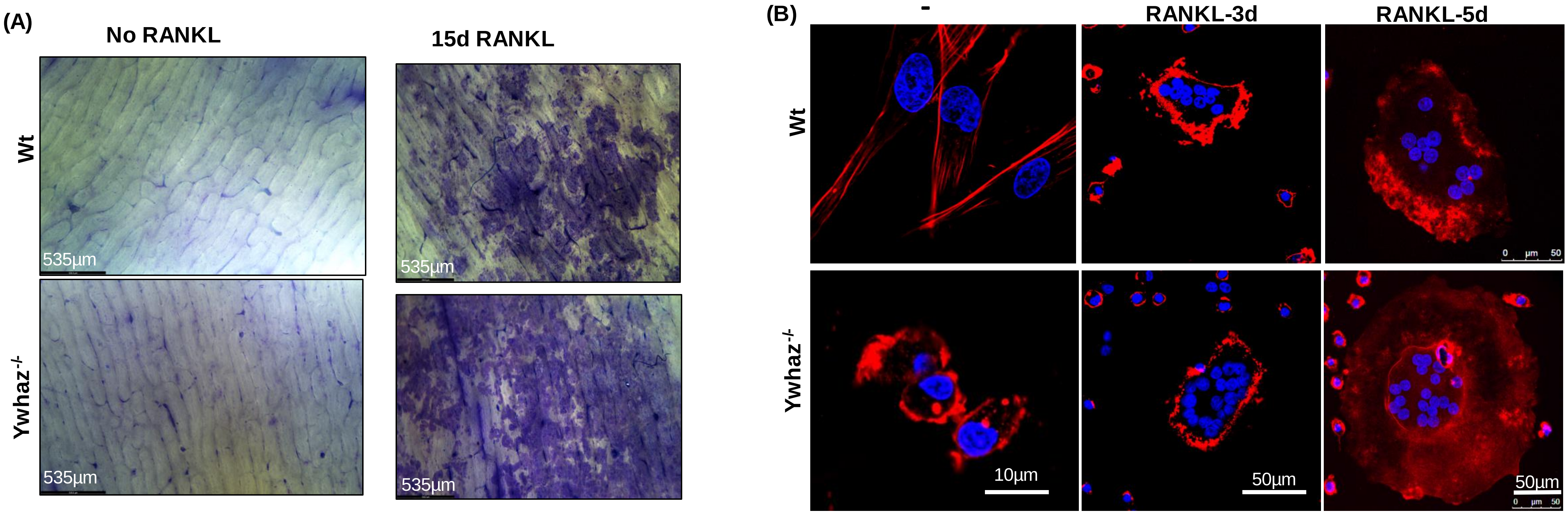

Supplementary Fig S1: (A) Wt and Ywhaz-/- BMDM were plated on bone disc for 0 and 15d were stained with toluidine blue. (B) Phalloidin stained Wt and Ywhaz$^{-/-}$ BMDMs, naïve or RANKL-treated, were imaged using confocal microscope. The scale bars are shown.

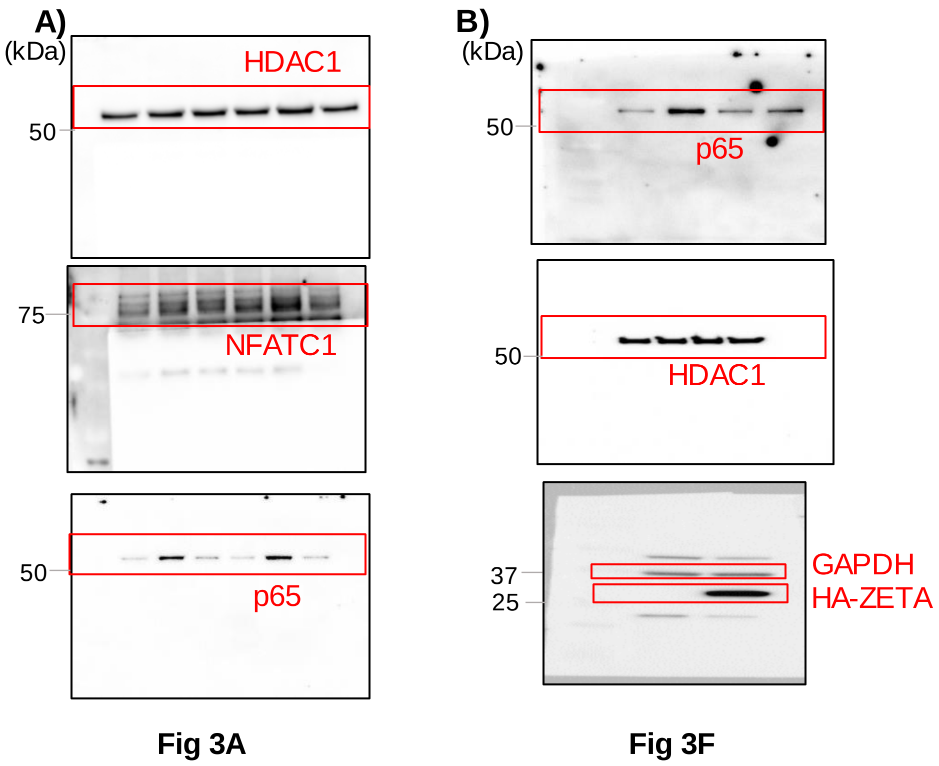

Supplementary Fig S2: (A-B) Raw data of Fig 3A and 3F. The blot showing NFATC1 was partly covered while imaging to keep focus on specific bands in correct MW range.

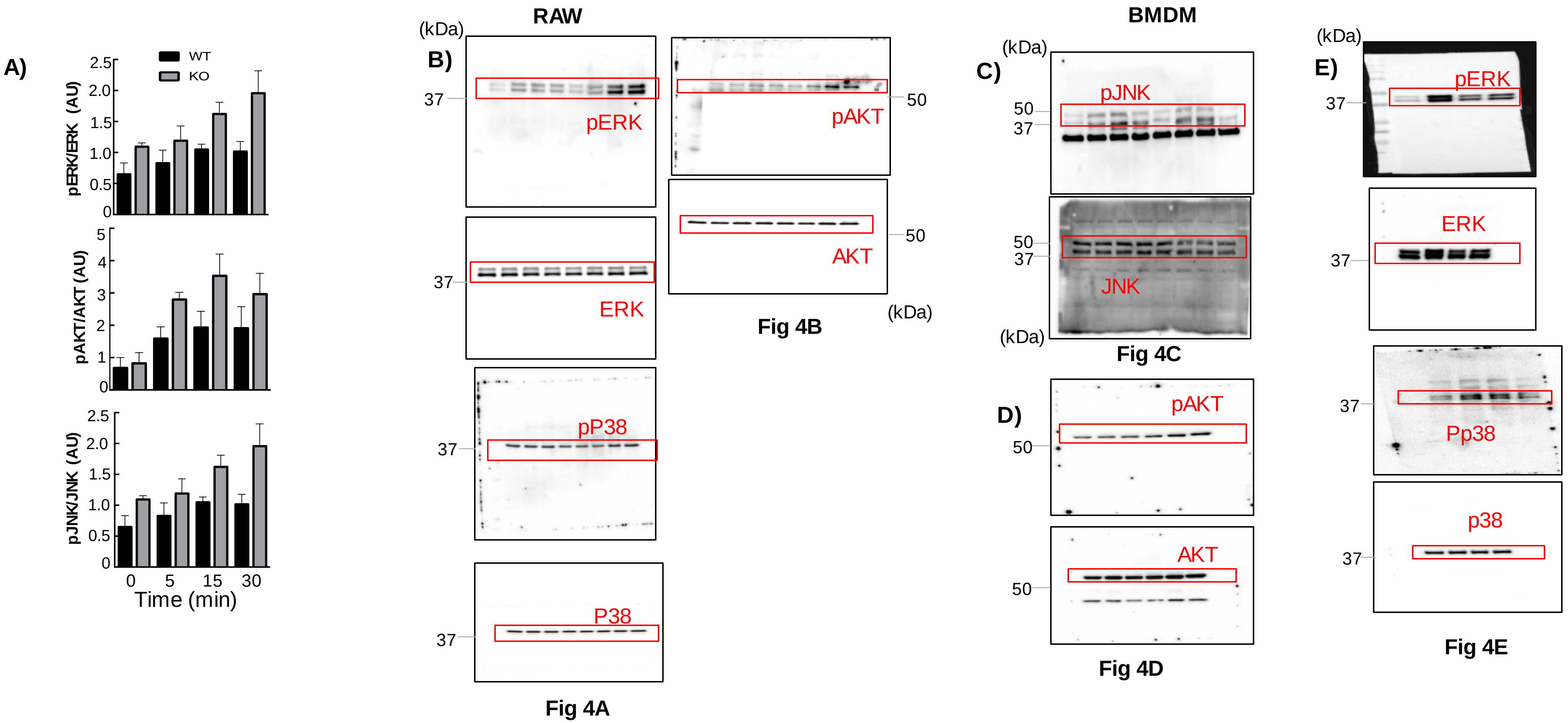

Supplementary Fig S3: (A) Quantitative analysis of phosphorylated kinases in RANKL-stimulated Ct and *Ywhaz*^{KO} RAW cells (n=3). (B) Raw data of Fig 4 (A-B). (C) Raw data of Fig 4C. (D) Raw data of Fig 4D. (E) Raw data of Fig 4E.

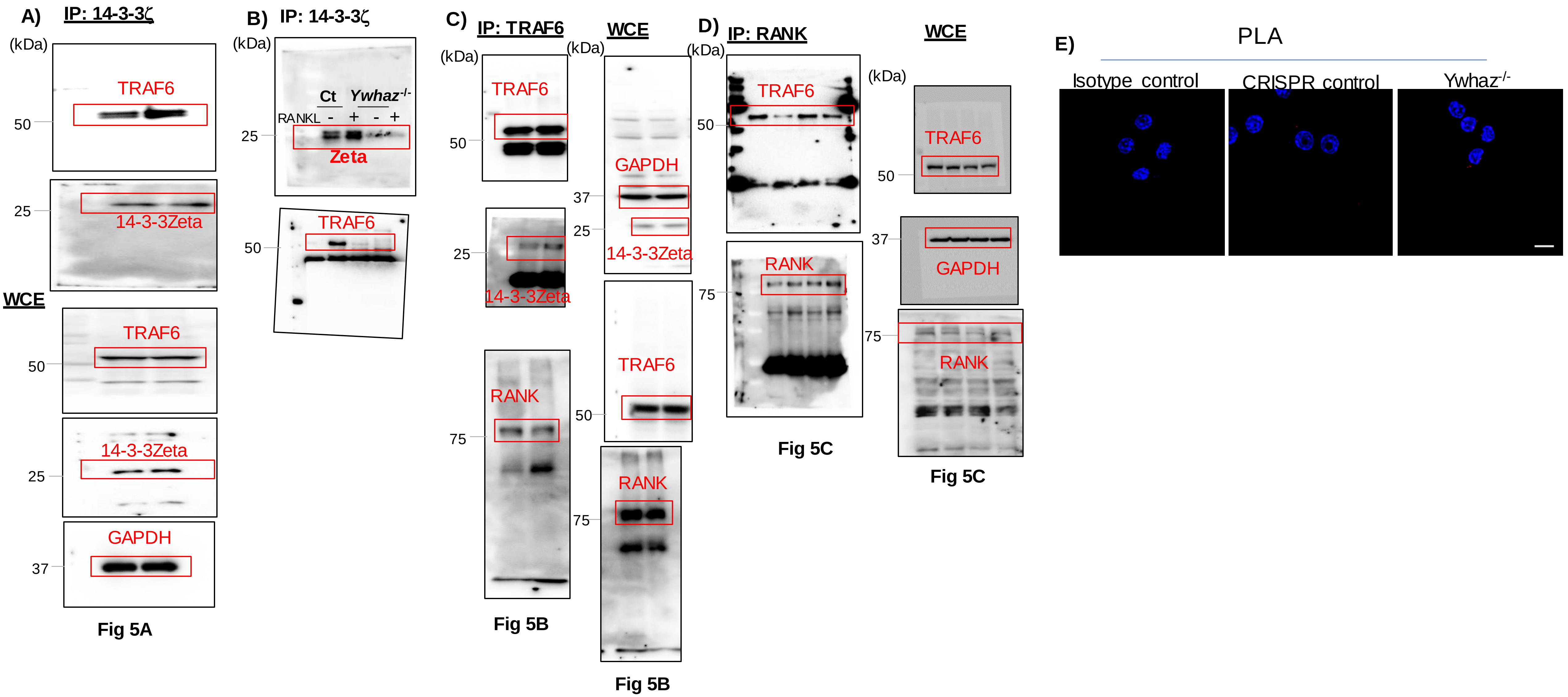

Supplementary Fig S4 : (A) Raw data of Fig 5A. (B) Effect of RANKL on 14-3-3ζ KO cells. (C) Raw data of Fig 5B. (D) Raw data of Fig 5C. (E) Additional PLA results showing TRAF6 and RANK interaction in Ct and *Ywhaz*^{KO} cells. The scale bar shows 10μm.

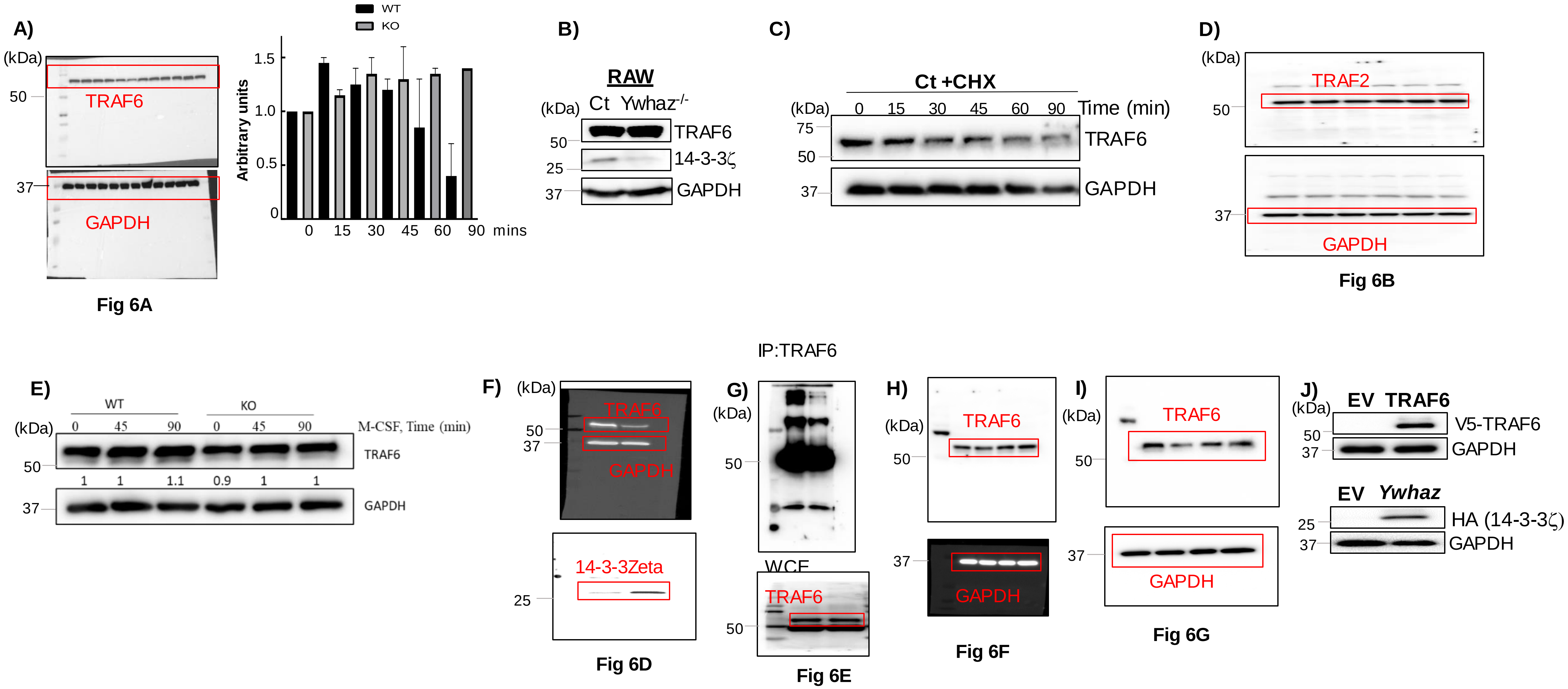

Supplementary Fig S5: (A) Raw data of Fig 6A and quantitative analysis of TRAF6/GAPDH ratio showing the effect of 14-3-3ζ on TRAF6 stability in RANKL-stimulated Ct and *Ywhaz*^{KO} cells. (B) TRAF6 levels at the basal level are comparable between Ct and *Ywhaz*^{KO} cells. (C) Effect of cycloheximide on TRAF6 stability in RANKL-treated Wt cells is shown. (D) Raw data of Fig 6B. (E) Effect of M-CSF on TRAF6 stability in Ct and *Ywhaz*^{KO} cells is shown. (F) Raw data of Fig 6D. (G) Raw data of Fig 6E. (H) Raw data of Fig 6F. (I) Raw data of Fig 6G. (J) Overexpression of V5-TRAF6 or HA-*Ywhaz,* compared to EV, in the Wt BMDMs are shown.
